# Gel-Based Proteomics of Clinical Samples Identifies Potential Serological Biomarkers for Early Detection of Colorectal Cancer

**DOI:** 10.3390/ijms20236082

**Published:** 2019-12-02

**Authors:** Stine F. Thorsen, Irina Gromova, Ib J. Christensen, Simon Fredriksson, Claus L. Andersen, Hans J. Nielsen, Jan Stenvang, José M.A. Moreira

**Affiliations:** 1 Institute of Drug Design and Pharmacology, Faculty of Health and Medical Sciences, University of Copenhagen, 2100 Copenhagen, Denmark; stinebt@gmail.com; 2 Danish Cancer Society Research Center, 2100 Copenhagen, Denmark; iig@cancer.dk; 3 Department of Surgical Gastroenterology, Hvidovre Hospital, University of Copenhagen, 2650 Hvidovre, Denmark; ib.jarle@finsenlab.dk (I.J.C.); h.j.nielsen360@gmail.com (H.J.N.); 4 Olink Bioscience, Uppsala Science Park, 752 37 Uppsala, Sweden; simon@genagon.com; 5 Department of Molecular Medicine, Aarhus University Hospital, 8200 Aarhus, Denmark; cla@clin.au.dk

**Keywords:** colorectal cancer, two-dimensional gel electrophoresis, early detection, plasma biomarkers, proximity extension assay

## Abstract

The burden of colorectal cancer (CRC) is considerable—approximately 1.8 million people are diagnosed each year with CRC and of these about half will succumb to the disease. In the case of CRC, there is strong evidence that an early diagnosis leads to a better prognosis, with metastatic CRC having a 5-year survival that is only slightly greater than 10% compared with up to 90% for stage I CRC. Clearly, biomarkers for the early detection of CRC would have a major clinical impact. We implemented a coherent gel-based proteomics biomarker discovery platform for the identification of clinically useful biomarkers for the early detection of CRC. Potential protein biomarkers were identified by a 2D gel-based analysis of a cohort composed of 128 CRC and site-matched normal tissue biopsies. Potential biomarkers were prioritized and assays to quantitatively measure plasma expression of the candidate biomarkers were developed. Those biomarkers that fulfilled the preset criteria for technical validity were validated in a case-control set of plasma samples, including 70 patients with CRC, adenomas, or non-cancer diseases and healthy individuals in each group. We identified 63 consistently upregulated polypeptides (factor of four-fold or more) in our proteomics analysis. We selected 10 out of these 63 upregulated polypeptides, and established assays to measure the concentration of each one of the ten biomarkers in plasma samples. Biomarker levels were analyzed in plasma samples from healthy individuals, individuals with adenomas, CRC patients, and patients with non-cancer diseases and we identified one protein, tropomyosin 3 (Tpm3) that could discriminate CRC at a significant level (*p* = 0.0146). Our results suggest that at least one of the identified proteins, Tpm3, could be used as a biomarker in the early detection of CRC, and further studies should provide unequivocal evidence for the real-life clinical validity and usefulness of Tpm3.

## 1. Introduction

Colorectal cancer (CRC) is the third most common cancer and the second most common cause of death from cancer. The burden of CRC is considerable: in 2018, an estimated 1.8 million new colorectal cancer cases and 881,000 deaths were expected to occur, accounting for about 1 in 10 cancer cases and deaths [1,2]. For the majority of patients, early-stage CRC is asymptomatic. As a result, most CRCs are first diagnosed at later, symptomatic, advanced stages that carry a much poorer prognosis. Approximately 40% of CRC patients are diagnosed with early, localized-stage disease (with 5-year survival rates of about 90%). However, most patients are diagnosed at advanced stages, and as a result, their 5-year survival rates decline to 70% (if presenting with local spreading) and 13% (if presenting with distant metastasis).

CRC is believed to progress gradually from surgically tractable precancerous lesions to highly malignant tumors; consequently, detection of the disease at the early stages is expected to reduce both the incidence and mortality of the disease, as the risk of recurrence and subsequent death due to CRC grows with increasing tumor stage at the time of primary diagnosis. One could then conceivably expect that a biomarker test of adequate diagnostic sensitivity and specificity would impact disease outcome. However, inherent problems such as overdiagnosis and lead time bias can restrict the usefulness of a biomarker for early cancer detection, and early diagnosis may not necessarily have an impact on survival. Evidence from multiple randomized trials convincingly demonstrated the effectiveness of screening asymptomatic individuals in reducing CRC incidence and mortality rates compared with no screening [3,4,5,6,7]. In addition, the implementation and analysis of national screening programs for CRC have established that early detection of CRC can significantly reduce the risk of death from CRC [8,9], making the case for early detection in CRC.

The most commonly used assay for CRC screening is the fecal occult blood test (FOBT), which uses immunochemical techniques to identify the presence of blood in stool. Although the FOBT test actually performs well, with biennial screening shown to reduce mortality from CRC by 15–21%, it suffers from a number of issues: it has a relatively low sensitivity, especially for detecting cancers located in the distal colon; it may be affected by certain foods, dietary supplements, and medications; and there is a considerable problem with patient compliance [10,11,12,13,14]. Direct imaging diagnostic technologies, such as colonoscopy and flexible sigmoidoscopy, have high specificity and sensitivity [15,16,17] and potentially can improve the detection rate of CRC; however, their primary diagnostic value is limited by their high-cost and invasiveness, the associated risk of bowel perforation, and the discomfort that it causes patients. Another emerging screening tool is multi-target stool DNA testing (MT-sDNA). MT-sDNA testing is a non-invasive assay approved by the US Food and Drug Administration for CRC screening that is based on the detection of an epigenetic signature associated with colorectal neoplasia [18]. Although MT-sDNA testing shows high sensitivity for CRC, it has a lower range of specificity than FOBT. In addition, it has lower sensitivity for the detection of advanced adenomas, which is essential for the effectiveness of any CRC population screening program. Therefore, there is still a crucial need for the identification of safe, cost-effective, minimally invasive biomarkers that can allow early detection of CRC with high sensitivity and specificity.

Numerous biomarker discovery studies have been conducted to address this need and identify CRC biomarkers in relevant biomaterials such as stool or serum [reviewed in [19]]. Proteomics methods are ideally suited to cancer biomarker discovery and a large number of studies have identified numerous deregulated proteins in CRC that could be candidate biomarkers [20,21,22]. Although, several potential biomarkers have been identified in this manner, they have, by and large, fallen short of the necessary specificity and sensitivity required to implement them in routine clinical use. One major challenge that has been repeatedly identified is the general lack of a coherent process connecting biomarker discovery and validation [23,24]. We tried to establish a coherent process for biomarker discovery in CRC that could increase the probability of identifying biomarkers of clinical value. Our biomarker discovery procedure was divided into three interconnected steps: (i) identification of deregulated proteins by a 2D gel-based analysis of a cohort composed of 128 CRC and 148 site-matched normal tissue biopsies, (ii) prioritization of identified candidate biomarkers based on presence in plasma, successful analytical assay development, and determination of ratios of upregulation in CRC samples, and (iii) first validation of identified biomarkers in an independent matched case-control set of plasma samples in four groups consisting of 70 patients each, including patients with CRC, adenomas, or non-cancer diseases and healthy individuals. Although 2D PAGE-based proteomics has fallen in disuse, there are still many inherent advantages to this technology (for an in-depth discussion, see [25,26]). Two of these were paramount for our decision to use 2D PAGE proteomics in this study: first, top-down quantitative analysis of intact proteins can be easily carried out on a large number of samples. Given that we used strict standard operating procedures for sample preparation and gel electrophoresis, the 2D gel patterns obtained are directly comparable to those from other studies. We have generated thousands of 2D gel patterns over the years, which dramatically decrease the workload associated with comparative studies. Second, 2D PAGE-based separation of proteins enables the identification of proteoforms, and we have previously identified proteoforms associated with neoplastic transformation [27]. Malignancy-associated proteoforms constitute excellent biomarker candidates. We describe our results here and discuss our efforts to establish a biomarker discovery pipeline, and the outcome of our approach as well as future initiatives.

## 2. Results

### 2.1. Biomarker Discovery

#### 2D Gel-Based Proteomic Profiling of Normal and Matched CRC Tissue Samples

In the discovery step, we employed a combination of two-dimensional (2D) gel electrophoresis (isoelectric focusing, IEF and nonequilibrium pH gel electrophoresis, NEPHGE) with image analysis (immunofluorescence) and mass spectrometry-based identification of differentially expressed proteins in colorectal adenocarcinomas compared to normal tissue samples. We chose this strategy based on a number of considerations: First, although 2D gel-based analysis has a number of drawbacks, the most relevant one in this context being the limitation of the technology to the more abundant proteins, it does have a number of advantages. Since we were interested in detecting tumors in the earliest possible stages of tumor growth, and given the correlation between blood biomarker levels and tumor volume/growth [28], it was reasonable to assume that the most abundant tumor-derived proteins will have the highest probability of being present at measurable concentrations after dilution in the circulation, even taking into account unfavorable shedding and clearance rates. Second, the top-down intrinsic properties of 2D gel-based proteomics would ease possible sample protein degradation issues, which are always a concern when dealing with CRC tissue samples. Third, complementing the 2D gel-based analysis of samples with immunofluorescence analysis would directly link candidate biomarker identification with assay development, as we could test antibody specificity by 2D analysis of the samples, where the deregulated proteins were originally identified. This approach combines the intrinsic sensitivity and cellular resolution of immunolocalization, with the specificity of 2D Western blotting and MS-based 2D gel protein identification [29].

We used freshly collected human colorectal tissue biopsies, tumor and normal, as the source of biological material due to their clinical relevance. We decided to analyze tissue biopsies, identify potential biomarkers, and develop targeted analytical assays that could be used for serum determinations, instead of analyzing blood (or serum or plasma) directly. Our decision was essentially based on the fact that potential biomarkers are present at exceedingly low levels in the blood as a result of dilution, and there is an extremely large dynamic range of protein abundance in these biofluids, making biomarker discovery a challenging prospect, particularly when using a 2D PAGE-based approach, which we chose for the reasons stated previously.

**Figure 1:**
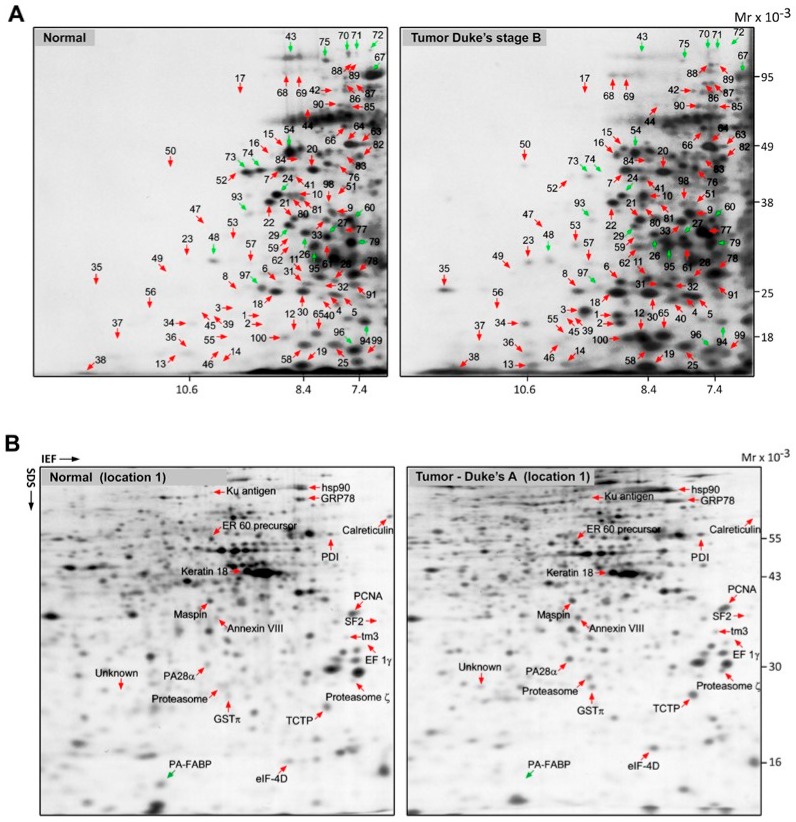
2D PAGE separation of whole protein extracts from normal and tumor tissues. (**A**) 2D PAGE (NEPHGE) separation of whole protein extracts from a normal tissue and a tumor tissue (Dukes’ B) (left and right panels, respectively). Gels were stained with silver nitrate and the identity of the various proteins was determined by mass spectrometry. The identity of the polypeptides indicated with numbers is given in Supplementary Table S2. (**B**) Protein lysates from a normal tissue and a tumor sample (Dukes’ A) were separated by 2D PAGE (IEF) and visualized with silver nitrate (left and right panels, respectively). The identity of the differentially expressed proteins was determined by mass spectrometry and is indicated for reference.

Tissue biopsies were divided and matched, according to Dukes’ stage of tumors as well as the sampling site. The site of sampling was recorded to ensure broad coverage, given that right- and left-sided neoplasms (proximal and distal to the splenic flexure) are considered distinct tumor entities, initiated by two different genetic mechanisms, microsatellite instability (MSI) and chromosomal instability (CIN), which contribute to carcinogenesis in the proximal and distal segments of the large bowel, respectively [30,31]. Proteome expression profiles of matched normal/tumor colon tissue pairs were analyzed using 2D PAGE in combination with mass spectrometry and database matching. Representative NEPHGE 2D gels for a normal sample (location 1) and a matched primary tumor (Dukes’ B, location 1) are shown in Figure 1A. For each case, differentially expressed proteins were pinpointed by image analysis and proteins recovered from silver-stained 2D gels were identified by MALDI-TOF-MS. A representative 2D IEF gel set of a normal sample (location 1) and its matched primary tumor (Dukes’ A, location 1) are shown in Figure 1B with differentially overexpressed proteins highlighted. In the combined NEPHGE and IEF analysis, we identified 63 consistently upregulated polypeptides (factor of four-fold or more, across multiple samples) that represented potential CRC biomarkers for early detection (Table 1).

## 2.2. Biomarker Exploratory Phase

### 2.2.1. Selection of Candidate Proteins

**Figure 2:**
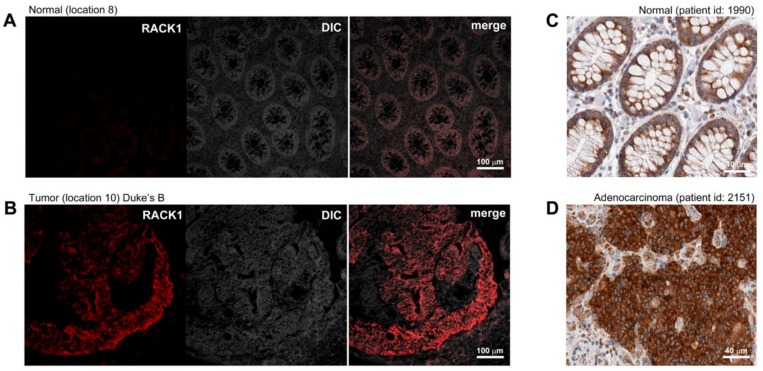
Indirect immunofluorescence analysis of differentially expressed proteins. Expression of Rack1, ascertained by immunofluorescence in representative (**A)** normal and (**B**) tumor frozen tissue samples included in this study as well as by immunohistochemistry of (**C**) normal and (**D**) tumor tissues (images retrieved from human protein atlas HPA; www.proteinatlas.org/pathology, last accessed 03.03.2019), was examined to ascertain the differential cell type-specific expression of this candidate biomarker. Magnification is provided in each case by scale bars (100 µm and 40 µm).

A comparison of the 2D protein profiles of the matched tumors and non-malignant tissues, illustrated in Figure 1A,B, identified 63 polypeptides that were consistently overexpressed in multiple tumor tissue biopsies and that were either absent or present at much lower levels in site-matched non-malignant tissues. We decided, in a first approximation, to select 10 out of the 63 upregulated polypeptides identified in the discovery phase for further assay development and validation. We ranked the hits according to their ratios of expression in CRC tissues compared to non-malignant tissues and incidence of deregulation in tumor samples ascertained by 2D gel-based analysis, and selected the first ten hits for which we could find reagents for assay development (Table 2). In order to confirm differential expression of the selected biomarkers at the cellular level, the expression of the putative CRC biomarkers was verified by immunofluorescence (IF) analysis of non-malignant and tumor samples (Table 3). Figure 2 shows representative IF pictures of tissue sections from a non-malignant tissue and a tumor tissue reacted with antibodies against Rack1 (parts A and B of Figure 2, respectively). As expected from the 2D gel-derived data, these markers were expressed at moderate to high levels by CRC cells, but at significantly lower levels in normal samples (Figure 2A,B). Overall, the results showed a suitable correlation between the gel-derived data and expression observed by IF, largely supporting the 2D gel results. Although immunostaining of samples allowed us to verify protein expression in the original tissue samples, the effect of fixation on staining intensity and antibody specificity are always concerns that should to be addressed. To do so, we complemented our analysis with publicly available data from the Human Protein Atlas (HPA) project. The HPA project created a map of protein expression patterns in normal cells, tissues, and cancer, and contains millions of images generated by immunohistochemically stained tissue sections with 26,009 antibodies directed toward 17,000 unique human proteins [32]. These images can be searched and examined for the expression profile of a given candidate protein in any given tissue (https://www.proteinatlas.org/). We compared the expression profiles of our 10 candidate biomarkers in the CRC samples available at HPA with our own immunostaining results. In all cases, we found an agreement between our results and the data available at HPA (Table 3, Figure 2).

### 2.2.2. Application to Human Plasma Samples for Verification of Clinical Utility

Having established the validity of the gel-based data, we proceeded with the validation phase. As the ultimate aim of our work was to identify potential biomarkers for a blood-based test, we performed an exploratory study to verify the presence of the 10 candidate biomarkers in human plasma. Because we expected these biomarkers to be present in low abundance in plasma, we chose to use the proximity extension assay (PEA) [33] and developed assays for each of the putative biomarkers that were identified. In a first step, we examined the technical discriminatory power of the developed assays. This was assessed by performing dose-response curves in a buffer with recombinant protein spike-ins and by assessing the sensitivity, specificity, and cross-reactivity for each of the assays ({Moreira, 2019 #239}). As a result, we excluded three assays that did not fulfill our technical requirements, leaving us with seven validated assays to test seven independent biomarkers (Table 2).

**Figure 3:**
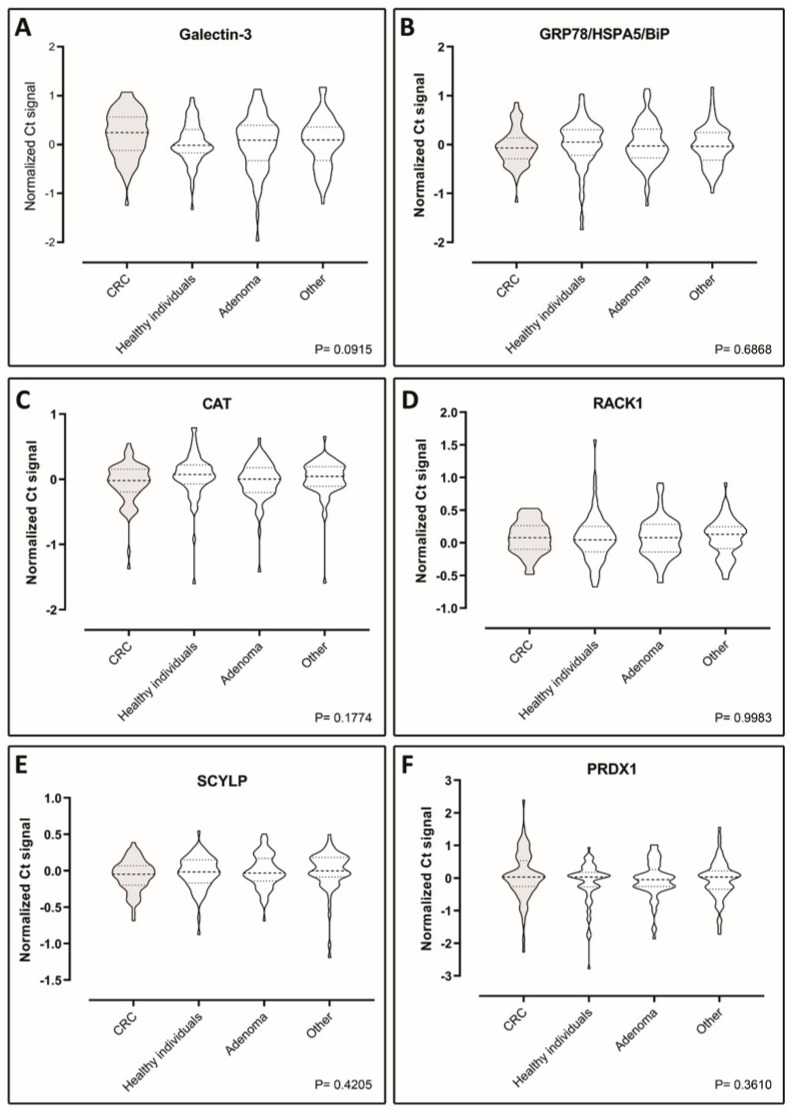
Violin plots showing comparisons of the plasma levels of six biomarkers—(**A**) galectin-3, (**B**) Grp78, (**C**) Cat, (**D**) Rack1, (**E**) Scylp, and (**F**) Prdx1—between CRC patients, healthy individuals, adenoma patients, and patients with diseases other than cancer (70 case-control sample set). Data are presented as Ct normalized signals measured for each protein in all four groups. The *p*-values were determined using ANOVA statistical tests. Mean values are indicated by horizontal bars. CRC sample plots are grey-shaded, all others are white.

**Figure 4:**
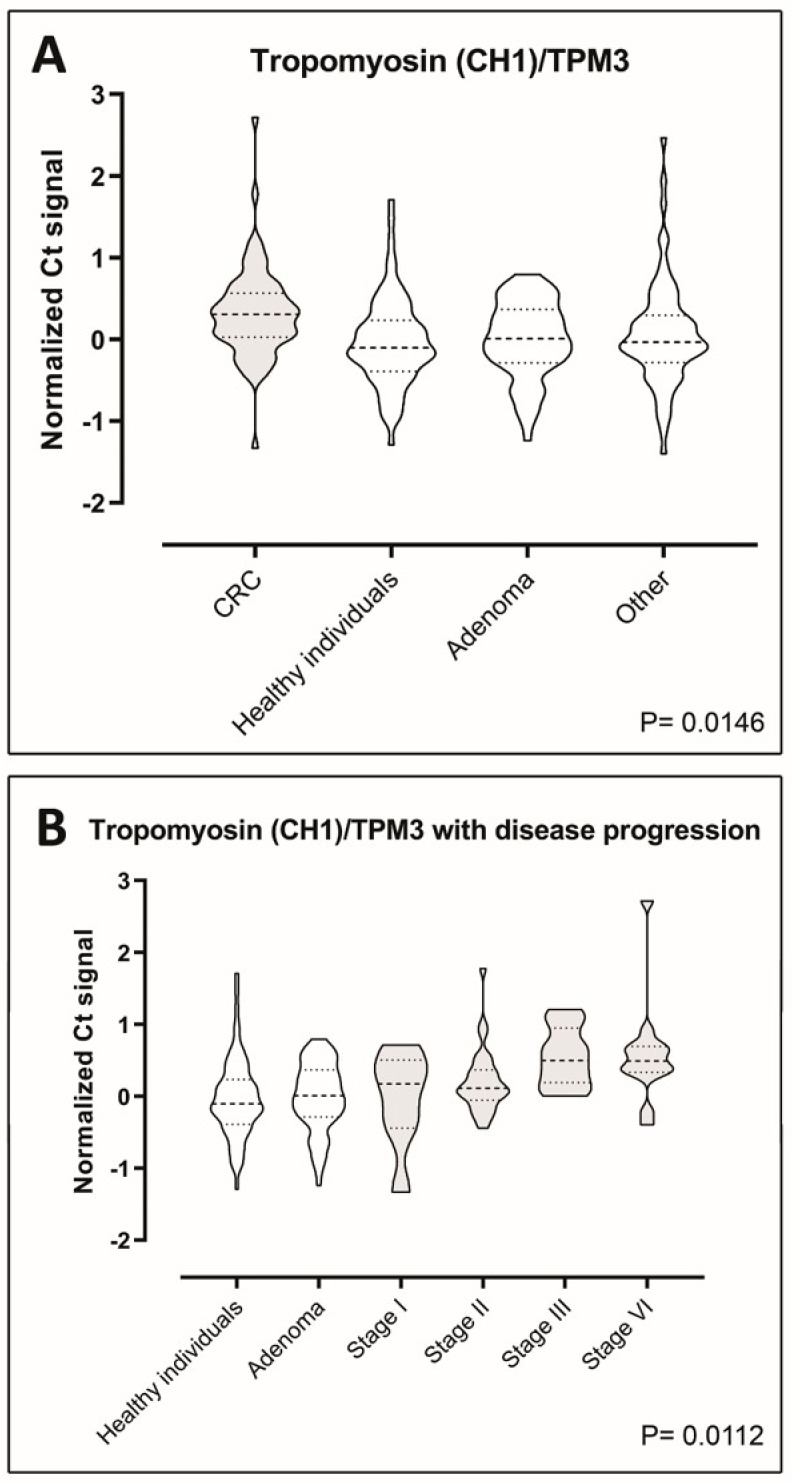
Tropomyosin-3 plasma levels. (**A**) Violin plots showing comparisons of the plasma levels of tropomyosin-3 between CRC patients, healthy individuals, adenoma patients, and patients with diseases other than cancer (70 case-control sample set). Data are presented as Ct normalized signals measured for each protein in all four groups. The *p*-values were determined using ANOVA. Horizontal bars indicate mean. (**B**) Violin plots showing changes of the plasma levels of tropomyosin-3 with disease progression. Cancer samples were divided according to the different stages of CRC patients (*n* = 70) (stages I through IV). Data are presented as Ct normalized signals measured for each protein in all stages. Mean values are indicated by horizontal bars. CRC sample plots are grey-shaded, all others are white.

The levels of the seven putative biomarkers were analyzed by PEA in plasma samples from a case–control study consisting of retrospectively sampled material including 70 healthy individuals with no pathological findings by endoscopy and/or no self-reported disease or medication, 70 adenoma patients, 70 CRC patients, and 70 patients with non-cancer diseases. We were able to detect all the seven proteins in all of the samples included in the study, demonstrating functionality of the assays (Figure 3A–F and Figure 4A) However, Tpm3 was the only discriminator of CRC on a significant level (*p* = 0.0146) (Figure 4A). To investigate the distribution of Tpm3 throughout CRC progression, we stratified results based on the Union for International Cancer Control’s (UICC) TNM tumor classification stages (Figure 4B). This demonstrated increased levels of Tpm3 as a function of increasing CRC stage (Figure 4B; *p* = 0.011).

## 3. Discussion

The purpose of this study was to develop a coherent gel-based proteomics biomarker discovery platform for the identification of clinically useful biomarkers for the early detection of CRC. To do so, we used 2D gel electrophoresis, a well-established and robust proteomic method, to analyze fresh CRC tissue biopsies in a first discovery phase, followed by a preclinical exploratory study and a validation study in plasma samples. The rationale was that this would provide a sufficient level of evidence for any biomarker that we discovered to be ready for preclinical development. Robustness and reliability were in focus throughout the process, and both biological and technical criteria were constantly reviewed in order to maintain stringency and thereby, improve the success rate of our candidate markers.

We analyzed tumor and site-matched normal tissues from patients with different tumor localizations and Dukes’ stage (Table 4 and Table 5, Figure S2), and identified 63 consistently upregulated polypeptides (factor of four-fold or more) in the combined NEPHGE and IEF analysis (Table 1). Several of these proteins, such as elongation factor 1-γ, profilin-1, annexin III, α-enolase, and Rack1, have previously been found in other studies to be associated with CRC [34,35,36,37]. We selected 10 out of the 63 consistently upregulated polypeptides that were identified (Table 2), and performed an exploratory study to verify the presence of the ten candidate biomarkers in human plasma. We established quantitative assays to measure the levels of each of the ten biomarkers in plasma, of which seven could be deployed. Following a first level analysis of the seven biomarkers, protein levels were analyzed by PEA in plasma samples from a cohort of 280 people, including healthy individuals with or without adenomas, CRC patients, and patients with non-cancer diseases (Figure 3 and Figure 4). One single protein, Tpm3, could discriminate CRC at a significant level (*p* = 0.0146) (Figure 4). Tpm3 is an actin-binding protein expressed in skeletal and smooth muscles and some non-muscular tissues. *TPM3* expression has been reported to control migration, invasion, and anchorage-independent growth of cancer cells by modifying the epithelial-mesenchymal transition pathway [38,39,40]. Moreover, a functional transformation screen performed with a cDNA library derived from a colorectal cancer biopsy has identified a chimeric “TPM3-TRK” fusion oncogene. This oncogene was the result of an intrachromosomal rearrangement that caused the fusion of the *TPM3* gene with a sequence encoding the transmembrane and intracellular domains of a transmembrane tyrosine kinase, TRK (tropomyosin receptor kinase) [41,42].

Tpm3 could be a useful diagnostic biomarker, particularly since one of the key requirements of a screening test for CRC is that it must allow detection of the disease at earlier stages so that the disease can be cured effectively. As a consequence, any candidate biomarker needs discriminatory power in the early stages of the disease, and Tpm3, according to our results can do so.

At this point, and from a mechanistic point of view, it is not entirely clear why some patients with CRC have increased levels of Tpm3 in their blood. However, a recent study found Tpm3 to be present in ovarian cancer patient sera at significantly elevated levels compared with controls [43], paralleling our own observation that Tpm3 is upregulated in CRC patient sera, and lending some support to further studies on the clinical usefulness of this protein. The Genotype-Tissue Expression (GTEx) project provides a comprehensive public resource to study tissue-specific gene expression and regulation. Samples collected from 53 non-diseased tissue sites across nearly 1000 individuals allow one to examine tissue-specific patterns of expression for any gene of interest. *Tpm3* mRNA expression in normal tissues was almost exclusively seen in skeletal muscle (Figure S1A). This is consistent with data available from HPA of immunohistochemistry (IHC) analysis of normal tissue samples, showing that the strongest expression of Tpm3 occurs in skeletal muscle samples (Figure S1B). In the case of normal colon samples, one can see occasional weak expression in glandular cells, and strong expression in the muscle cells of the lamina muscularis mucosae (Figure S1C, yellow arrow) as well as in vessels (Figure S1C, red arrow). In tumor samples, both stromal cells (Figure S1D,E, yellow arrows) and tumor cells (Figure S1D,E, black arrows) can express Tpm3 at different levels. The increased presence of Tpm3 in the sera of patients bearing CRC samples could then be the result of increased expression of Tpm3 in tumor cells, a reflection of invasion of tumor cells through the lamina muscularis mucosae, or a combination of both. Taken as a whole, our data suggest that upregulation of Tpm3 is a cancer-related event, and underscores the need for further studies to assess the clinical validity and utility of this protein as a biomarker for the early detection of cancer.

## 4. Materials and Methods

### 4.1. Biological Material

#### 4.1.1. Patient Sample Collection and Handling for 2D PAGE Analysis

Colorectal adenocarcinomas and site-matched normal tissue biopsies were collected from various locations in the colon and rectum, and defined as follows: location 1 (cecum), 2 (ascending colon), 3 (hepatic flexure), 4 (transverse colon), 5 (splenic flexure), 6 (descending colon), 7 (sigmoid colon), 8 (above peritoneal fold), 9 (at peritoneal fold), 10 (under peritoneal fold), and 11 (anal canal infiltration) (Table 4 and Table 5, Figure S2). Tumors were classified by Dukes’ staging. One hundred and twenty-eight tumor biopsies were collected at the Skejby Hospital, Aarhus, during a period of 3 years. The surgical guidelines implemented in Denmark stipulate that 5 cm of normal bowel on either side of the primary tumor are taken to minimize anastomotic recurrences. One hundred and forty-eight normal mucosa specimens were collected from the resection margins as distal to the tumor as possible (>2 cm and <5 cm). The project was approved by the Scientific Ethics Committee for Aarhus municipality. Tumors and normal biopsies, clean of clots and contaminating tissue, were minced into small pieces with the aid of a scalpel and were labeled with [^35^S]-methionine for 14–16 h in a 10 mL sterile plastic conical tube containing 0.2 mL of modified Eagle’s medium lacking methionine and containing 2% dialyzed (against 0.95% NaCl) fetal calf serum (FCS) and 100 µCi of [^35^S]-methionine (GE Healthcare Life Sciences, Amersham, UK). At the end of the labeling period, the medium was removed and protein extracts were prepared by tissue lysis in 0.3–0.4 mL of lysis solution [44,45]. Samples were stored at –20 °C until use. A pulsing resected tissue with radiolabeled methionine has a number of advantages: it considerably increases sensitivity, which is a limitation of 2D PAGE-based proteomics, but it also directs visualization to a newly translated protein in the freshly resected tissues, thus reducing the “noise” from background proteins present in the cells from the micro-environment and in the surrounding matrix.

#### 4.1.2. Patient Plasma Sample Collection and Handling for Protein Extension Analysis

Subjects undergoing a sigmoidoscopy or colonoscopy, either following symptoms consistent with CRC or after attending surveillance programs due to hereditary CRC (Hereditary nonpolyposis colorectal cancer and familial adenomatous polyposis), were included in this cross-sectional study. A total of 5165 subjects gave oral and written consents according to the Helsinki II Declaration. The study was approved by The Regional Ethical Committee of Greater Copenhagen and Frederiksberg, Denmark (KF 01-080/03; approval date: 01.12.2003). For the first level verification of protein biomarkers in plasma, a case-control study was designed by randomly picking 70 stage I–IV CRC patients from the larger cohort [28]. Matching was then done, based on age and gender and the following three groups of 70 cases each were selected; healthy individuals with no pathological findings by endoscopy and/or no self-reported disease or medication, adenoma patients, and patients with non-cancer diseases. Main clinical characteristics of the CRC patients included in the study are presented in Table 6. EDTA blood samples were collected from all subjects at the time of endoscopy following a strict standard operating procedure [46]. The samples were centrifuged at 2500× *g* for 10 min at 4 °C. After centrifugation, the plasma was aspirated, aliquoted, and stored at –80 °C until further analysis.

### 4.2. Two-Dimensional PAGE

2D PAGE was performed essentially as described [47] by using both isoelectric focusing (IEF; using carrier ampholytes) and non-equilibrium pH gradient electrophoresis (NEPHGE). Proteins were visualized by autoradiography and/or silver staining according to published procedures [48,49]. For quantitation, 2D gel autoradiographs were imaged using a Molecular Imager device (Bio-Rad Laboratories, Hercules, CA, USA) and images were analyzed using the PDQuest software (v.7.1; Bio-Rad Laboratories, Hercules, CA, USA). Gels were done in triplicate to minimize gel-to-gel variation. Only gels presenting well-focused spots and a limited amount of protein remaining at the origin were selected for quantitation. Protein levels were normalized to the levels of actin (IEF) and annexin II, which migrated in both IEF and NEPHGE gels. 

### 4.3. Mass Spectrometry

#### 4.3.1. In-Gel Digestion of Proteins Separated by Gel Electrophoresis 

Proteins were excised from silver-stained 2D gels and in-gel digestion was performed as previously described [48]. The excised gel plugs were washed in 50 mM NH_4_HCO_3_/acetonitrile (60/40) and dried by vacuum centrifugation, followed by reduction and alkylation of the proteins. Gel pieces were rinsed several times in 50 mM NH_4_HCO_3_ and dried by vacuum centrifugation. Trypsin (sequencing grade; Boehringer-Ingelheim, Ingelheim am Rhein, Germany), dissolved in 50 mM NH_4_HCO_3_ (12 ng/µL), was added to the dry gel pieces and reswelling was performed on ice for 1 h. The extra fluid was removed and 2–5 µL of digestion buffer was added. Samples were digested at 37 °C for 4–18 h. The peptide mixture was analyzed either directly by MALDI-MS or the peptides were additionally extracted from the gel piece with 50 µL of 60% acetonitrile and ultrasonication. 

#### 4.3.2. Sample Preparation 

Two MALDI sample preparation methods were used: (i) The co-crystallization of analytes without extraction with matrix was achieved by depositing a 0.5–1.0 µL 1:1 mixture of peptides from the supernatant in 2% trifluoroacetic acid with the matrix solution (15–20 g/L of α-cyano-4-hydroxycinnamic acid in 70% acetonitrile) directly on the MALDI target. (ii) The extracted peptides were concentrated on a C18 reversed-phase microcolumn integrated in the outlet of a P10 pipetting tip (Zip-Tip C18, Millipore, Burlington, MA, USA) according to the protocol recommended by the manufacturer. The peptides bound to the column were eluted with 0.2 µL of matrix solution (15–20 g/L of α-cyano-4-hydroxycinnamic acid in 70% acetonitrile) directly onto the MALDI target. 

#### 4.3.3. Peptide Mass Mapping by MALDI-MS 

A Bruker BIFLEX model MALDI-TOF mass spectrometer (Bruker-Franzen Analytik GmbH, Bremen, Germany) equipped with a Scout source and variable detector bias gating was used in positive-ion reflector mode for the mass analysis of peptide mixtures (peptide mass mapping). The ion acceleration voltage was set to 18.5 kV and the reflector voltage to 20 kV. Internal spectra calibration was performed using the tryptic autodigested peaks. Protein identification by peptide mass fingerprinting was performed by searching the peptide masses in a comprehensive non-redundant protein sequence database (OWL) encompassing SWISS-PROT, PIR, GenBank (translation), and NRL-3D. SWISS-PROT is the highest priority source, all others being compared against it to eliminate identical and trivially different sequences. For the interpretation of peptide mass maps and protein identification of protein digests, we used the UCSF Mass Spectrometry database search program MS-Fit (http://rafael.ucsf.edu/cgi-bin/msfit) and the Mascot Search Peptide Mass Fingerprint program developed by Matrix Science (http://www.matrixscience.com/cgi). The following settings were applied: relevant species is *Homo sapiens*, no molecular or pI restrictions, one missing cleavage possible, cysteine residues are modified, protein N-terminus is acetylated, and the mass tolerance was 10–20 ppm. Score cut-offs were determined for each replicate run, as previously described [50]. 

### 4.4. Indirect Immunofluorescence Analysis 

Tumors and normal biopsies, frozen in liquid nitrogen, were sectioned on a cryostat. The biopsy sections (8 µm in thickness) were placed in cover slips, washed 3 times with Hank’s buffered saline (HBSS), and treated for 10 min with 3.6% formaldehyde. After washing extensively with HBSS, the cover slips were covered with the primary antibody and incubated for 45 min at 37 °C in a humid environment. The cover slips were then washed several times with HBSS and covered with a rhodamine-conjugated relevant secondary antibody (diluted 1:50 in HBSS). After incubation for 45 min at 37 °C in a humid environment, the cover slips were washed thoroughly with HBSS and mounted in a fluorescence mounting medium (DAKO, Glostrup, Denmark). Sections were imaged using a Zeiss LSM510Meta confocal laser scanning microscope (Carl Zeiss MicroImaging GmbH, Jena, Germany). The standardization of dilution, incubation, and development times appropriate for each antibody allowed an accurate comparison of expression levels in all cases. Normal rabbit or mouse sera, instead of the primary antibody, were used as a negative control.

### 4.5. Proximity Extension Analysis (PEA) Validation Analysis

#### 4.5.1. Quality Criteria for Antibody Assessment

Affinity-purified polyclonal antibodies raised against the whole native recombinant protein are preferred when producing PEA probes. Most importantly, antibodies raised against a synthetic peptide cannot be used. Detailed information about the antibodies and antigen sources used in this study can be found in Table S1.

#### 4.5.2. Establishment of the PEA Probes

The PEA (A) and PEA (B) proximity probes were produced by covalently connecting a batch of an affinity-purified antibody to either a free 3′-hydroxyl or free 5′-phosphate oligonucleotide. These antibody-oligonucleotide conjugates were generated by Innova Biosciences (Cambridge, UK) using their Lightning-Link^TM^ technology. Conjugation quality was analyzed by SDS-PAGE (data not shown). The PEA (A) oligonucleotide sequences comprised a 40-mer 3′ and 5′ oligonucleotide sequences consisting of a central 20 bp universal sequence and a flanking 20 bp unique sequence for primer targeting in PCR amplification and qPCR [45,51]. The central 20 bp universal sequence was used as the hybridization site in the extension procedure and molecular beacon target in qPCR. The extension primer was hybridized to the 5′-free oligonucleotide of one of the proximity probe conjugates, at a 2:1 oligo-to-antibody ratio [19]. The connector oligonucleotide, the oligonucleotide pair sequences, forward primers (PreAmp-Frw, qPCR-Frw), and reverse primers (PreAmp-Rew, qPCR-Rew) were obtained from Biomers (GmbH, Ulm, Germany).

#### 4.5.3. Proximity Extension Assay (PEA)

The protocol has previously been described in detail [20]. In brief, 1 µL of PBS + 0.1% BSA ± antigen spike-in or human EDTA plasma were mixed with 0.64 µL probe mix (50 pM of each PEA probe pair and internal control standard spike-in mix (GFP-labelled, extension oligonucleotide)) and 2.36 µL plasma dilution (Olink Bioscience, Uppsala, Sweden). After overnight incubation at 4 °C, tubes containing the 4 µL probed samples were transferred to a thermal cycler set at 37 °C and 76 µL of pre-extension mix (Olink Bioscience) was added, followed by incubation at 37 °C for 5 min. Subsequently, 20 µL of extension mix (Olink Bioscience) was added and the extension reactions were then run at 37 °C for 20 min followed by a 10 min heat inactivation step at 80 °C. 

Pre-amplification was performed in a total volume of 25 μL by mixing 20 μL of the ligated product with 5 μL PCR mix (1× PCR buffer (Invitrogen, Carlsbad, CA, USA), 15 mM MgCl_2_ (Invitrogen), 1 mM dNTP (Invitrogen), 0.2 µM of each forward and reverse pre-amplification primer (Table S2), and 7.5 units Platinum Taq polymerase (Invitrogen)) using the same amplification protocol as previous described [30]. Prior to real-time PCR, the products were diluted 5-fold in 1× Tris-EDTA buffer.

#### 4.5.4. Real-Time qPCR

The qPCR reactions were performed either on an ABI 9700 HT Fast (Applied Biosystems, Foster City, CA, USA) instrument using a 384-well format or the BioMark^TM^ microfluidic system (Fluidigm, San Francisco, CA, USA). Regardless of the instrument used, each protein assay used separate qPCR reactions with individual primer pairs (Frw and Rew qPCR primers, see Table S2). For the 9700 HT Fast system, the diluted DNA products were incubated for 30 min at 37 °C in a mixture containing 1.4X Fast Universal Master Mix (Applied Biosystems), dH_2_O, and 0.05 units of Uracil-DNA Excision Mix (Epicenter, Madison, WI, USA) and incubated for 30 min at 37 °C to digest any leftover primers in the solution and thereby reduce interference during qPCR. The qPCR reaction was performed by transferring 7 μL of the pre-amplified sample mix and 3 μL of 3 μM primer (Biomers, Ulm, Germany), dH_2_O, and 0.8 μM TaqMan probe (Applied Biosystems, Foster City, CA, USA) to a total sample volume of 10 μL per well. The thermal cycler program was initiated with 5 min at 95 °C followed by 45 cycles for 15 s at 95 °C and for 1 min at 60 °C.

### 4.6. Statistical Analysis 

Candidate markers for analysis were selected from the available molecular markers by choosing those with deregulated expression in greater than 30% of cases. The chosen markers were analyzed by a logistic regression analysis adjusting for the case-control design with CRC versus adenomas, non-neoplastic diseases, and healthy individuals. In all cases, *p*-values lower than 0.05 were considered significant. All calculations were performed using SAS (SAS Institute, Cary, NC, USA).

## 5. Conclusions

We implemented a coherent gel-based proteomics biomarker discovery platform for the identification of clinically useful biomarkers for early detection of CRC. Potential protein biomarkers were identified by 2D gel-based analysis of CRC samples and site-matched normal tissue biopsies. Identified putative biomarkers were prioritized and we developed analytical assays to quantitatively measure their presence in plasma. Our results suggest that at least one of the identified proteins, Tpm3, could discriminate CRC at a significant level (*p* = 0.0146), based on its serum levels. Further studies are warranted to establish clinical validity and usefulness of Tpm3.

## Figures and Tables

**Table 1:** Upregulated polypeptides (NEPHGE and IEF combined).

Identity	Apparent Mw (kDa)	Apparent pl	Sample Frequency ^a^ (Dukes’)				
			A	B	C	D	All Tumors
40S Ribosomal protein s10	17	10.3	0/1	4/14	2/9	1/7	7/31
Annexin II	38.5	7.9	1/1	7/14	3/9	5/7	16/31
Annexin VII	37.5	5.6	1/2	1/4	-	2/2	7/12
Calreticulin ^b^	60.7	4.2	2/2	4/4	-	2/2	11/12
Calreticulin ^b^	60.7	4.2	2/2	4/4	-	2/2	11/12
Catalase	66	7.9	0/1	7/14	2/7	2/7	11/29
Cofilin	19	8.2	2/3	9/12	4/8	7/8	22/31
Cyclophilin A ^b^	18.2	8	1/1	12/14	6/9	6/7	25/31
Cyclophilin A ^b^	18.1	8.3	0/3	11/11	7/9	7/8	25/28
Cyclophilin B ^b^	19.8	10.2	1/1	7/14	6/9	5/7	19/31
Cyclophilin B ^b^	19.1	10.2	1/1	9/14	5/9	3/7	18/31
EF-1ϒ	33	4.3	1/2	3/4	-	2/2	8/10
Eukaryotic translation initiation factor 5A-1 (eIF-4D)	16	4.9	2/2	3/4	-	2/2	9/11
Elongation factor 1 alpha (EF-1α)	19.7	9.9	4/6	16/19	16/18	7/8	43/51
Eukaryotic translation elongation factor 2 (EF-2)	94.3	6.4	1/2	3/3	-	1/1	8/9
F1 ATPase, alpha-subunit	53.8	7.6	0/1	4/14	4/9	2/7	10/31
Fructose biphosphate aldolase A	43.4	8.8	1/3	5/11	4/8	4/8	14/30
Fumarate hydrotase	44.2	7.9	1/1	11/14	6/9	5/7	23/31
FUSE binding protein (FBP, myc far upstream element-binding protein)	67.5	7.6	2/3	10/12	9/10	7/7	28/32
FUSE binding protein 2 (FBP2, KH-type splicing regulatory protein KSRP mRNA)	75	7.5	2/3	11/12	6/10	6/7	25/32
FUSE binding protein 3 (FBP3)	63.4	8.2	0/1	10/14	5/9	3/7	18/31
Galectin-3	26.1	8.6	1/1	6/14	4/9	5/7	16/31
Glutamate dehydrogenase	53.4	7.9	1/1	11/14	9/9	6/7	27/31
Glyceraldehyde 3-phosphate dehydrogenase (GAPDH) ^b^	19.1	8.9	4/6	16/19	16/18	7/8	43/51
Glyceraldehyde 3-phosphate dehydrogenase (GAPDH) ^b^	19.9	8.9	4/6	16/19	16/18	7/8	43/51
Endoplasmic reticulum chaperone BiP (GRP78)	75.5	4.9	-	2/4	-	1/2	6/10
Glutathione S-transferase P (GSTπ)	25.4	5.5	1/2	2/4	1/2	1/2	10/13
Heterogeneous nuclear ribonucleoproteins A2/B1 (hnRNP A2/B1)	38.9	8.3	1/1	6/14	4/9	4/7	15/31
*Homo sapiens* DNA-binding protein (CROC-1B)	19	7.1	0/1	11/14	6/9	4/7	21/31
HSP47 (collagen-binding protein 2)	48.6	9.3	2/3	12/12	9/9	7/8	30/32
Heat shock protein 90 (Hsp90)	89.5	4.9	2/2	1/4	-	2/2	6/11
IG gamma-1 chain C region	67	7.6	1/1	14/14	7/9	7/7	29/31
Isocitrate dehydrogenase	46.8	7	0/1	7/14	2/7	2/7	11/29
Keratin 18	43.6	5.3	3/3	3/4	1/1	1/2	9/12
Ku antigen (86 kDa)	85.6	5.7	3/3	2/4		1/2	8/11
Leukocyte elastase inhibitor	17	7.3	0/1	5/14	5/9	2/7	12/31
Maspin	38.7	5.8	3/3	3/3	-	0/2	8/11
Monocarboxylate transporter 1 (MCT-1)	19.1	10.2	1/1	2/14	5/9	4/7	17/31
Nucleoside diphosphate kinase B (NDK B)	19	8.7	2/3	11/12	9/10	5/7	27/32
Proteasome activator complex subunit 1 (PA28α)	30.4	5.8	3/3	4/4	-	1/2	10/12
Proliferating cell nuclear antigen (PCNA)	37.4	4.4	2/3	2/3	2/2	2/2	9/12
Peroxiredoxin-1	22	8.2	2/3	13/13	9/10	8/8	32/34
Phosphoglycerate kinase 1	43.6	8.2	1/1	9/14	4/7	6/7	20/29
Porin 2	35.3	7.9	2/3	9/11	7/10	4/8	22/32
Profilin-1	15.8	8.4	1/1	10/14	7/9	5/7	23/31
Proteasome	26.9	5.6	-	2/4	-	1/2	6/12
Proteasome C3	26.5	7.2	1/1	6/14	6/9	4/7	17/31
Proteasome Ϛ	28.1	4.4	2/3	2/4	1/1	2/2	11/14
Protein disulfide isomerase (PDI)	56.2	4.6	2/2	4/4	1/1	2/2	11/12
Protein disulfide isomerase ER 60 precursor	56.5	5.7	2/2	-	-	1/2	9/11
Putative secreted protein XAG2	17.3	10	1/1	10/14	4/9	3/7	18/31
Receptor of activated protein C kinase 1 (Rack1)	32.5	8.3	3/3	9/11	6/10	3/8	21/32
Raf kinase inhibitor protein (RKIP)	23	8.2	1/1	11/14	4/9	7/7	23/31
Smooth muscle protein 22-alpha (SM-22α)	24.9	8.3	1/3	8/10	2/6	6/8	17/27
Sorbitol dehydrogenase	42.4	8.3	1/1	10/14	7/9	3/7	21/31
Splicing factor SF2 p32 subunit	35.5	4.2	1/1	4/4	-	2/2	10/10
Thyroid hormone receptor-interacting protein 1 (26S protease regulatory subunit 8)	46.8	7.1	1/1	12/14	6/7	6/7	26/31
Translationally controlled tumor protein (TCTP)	24.2	4.6	0/2	2/4	0/1	2/2	6/12
Triosephosphate isomerase	28.3	7.3	1/1	11/14	7/9	5/7	24/31
Tropomyosin-3 (tm3)	33.7	4.4	2/3	2/4	-	2/4	8/10
Vinculin (metavinculin)	65	7.5	1/1	13/14	4/9	7/7	25/31
α-Enolase	49.2	7.5	1/1	11/14	4/7	5/7	21/29

^a^ Frequency denotes the number of samples where the protein was found upregulated relative to the number of total samples where the protein was identified. ^b^ Non-unique proteins, multiple spots correspond to the same protein identity.

**Table 2:** Markers selected for verification in the exploratory study.

Protein Name	Symbol	UniProtKB	Deregulation Frequency ^c^
78 kDa glucose-regulated protein ^a,b^	Bip/Grp78/HspA5	P11021	6/10
α-Enolase ^a^	Eno1	P06733	21/29
Catalase ^a,b^	Cat	P04040	11/29
Cyclophilin B ^a,b^	Scylp/PPIase B/PPIB	P23284	18/31
Eukaryotic translation elongation factor 2 ^a^	eEF2/EF-2	P13639	8/9
Galectin-3 ^a,b^	Gal-3	P17931	16/31
Peroxiredoxin-1 ^a,b^	Prdx1/Nkef-A	Q06830	32/34
Profilin-1 ^a^	Pfn1	P07737	23/31
Receptor for activated C kinase 1 ^a,b^	Rack1	P38011	21/32
Tropomyosin alpha-3 chain ^a,b^	Tpm3/tm3	P06753	8/10

^a^ Markers for which PPA probes and corresponding assays were developed. ^b^ Markers that reached the validation phase. ^c^ Frequency denotes the number of samples where the protein was found upregulated relative to the number of total samples where the protein was identified.

**Table 3:** Immunolocalization and expression of candidate markers in CRC samples.

Protein	Expression in CRC Samples	HPA Concordance (Reference Antibody)
Grp78	Expression in tumor cells, with samples varying from weak to very strong expression	Yes (HPA038845)
Eno1	Expression in tumor cells, with samples varying from not detected to strong expression	Yes (CAB018614)
Cat	Some samples showed low to moderate expression in tumor cells	No (HPA051282)
PPIB	Expression in tumor cells, with samples varying from not detected to strong expression	Yes (HPA012720)
eEF2	Ubiquitous strong expression in tumor cells	Yes (HPA040534)
Gal-3	Expression in tumor cells, with samples varying from weak to very strong expression	Yes (HPA003162)
Prdx1	Expression in tumor cells, with samples varying from weak to very strong expression	Yes (CAB004682)
Pfn1	Some samples showed low to moderate expression in tumor cells	Yes (CAB037134)
Rack1 ^a^	Medium to strong expression in tumor cells, with samples varying from not detected to strong expression	Yes (CAB004288)
Tpm3 ^b^	Strong expression in skeletal muscle and endothelial cells. Tumor cells showed low to moderate expression in tumor cells	Yes (HPA009066)

^a^ Depicted in Figure 2. ^b^ Presented in Figure S1.

**Table 4:** Distribution of tumor biopsies according to Dukes’ stage and location.

Dukes’ Stage	Loc. 1	Loc. 2	Loc. 3	Loc. 4	Loc. 5	Loc. 6	Loc. 7	Loc. 8	Loc. 9	Loc. 10	Loc. 11	Total
A	2	2	1	0	0	1	2	2	1	3	1	15
B	8	6	0	1	3	1	14	2	7	7	0	49
C	6	2	1	1	1	1	10	8	1	17	0	48
D	1	0	1	0	0	1	8	1	2	2	0	16
Total	17	10	3	2	4	4	34	13	11	29	1	

**Table 5:** Distribution of normal tissue biopsies according to location and Dukes’ stage of the adjacent tumor.

Dukes’ Stage	Loc. 1	Loc. 2	Loc. 3	Loc. 4	Loc. 5	Loc. 6	Loc. 7	Loc. 8	Loc. 9	Loc. 10	Loc. 11	Total
A	2	2	1	0	0	2	1	2	1	4	1	16
B	9	5	0	1	3	1	16	2	7	7	0	51
C	7	3	1	1	2	1	9	9	2	18	1	54
D	1	0	1	0	0	1	8	1	4	2	0	18
No cancer	3	1	0	2	0	0	1	1	1	0	0	9
Total	22	11	3	4	5	5	35	15	15	31	2	

**Table 6:** Clinical characteristics for the 70 CRC patients included in the study.

		*Subjects, n (%)*
*Gender*		
Female		35 (50)
Male		35 (50)
*Age group*		
40–49		3 (4)
50–59		9 (13)
60–69		17 (24)
70–79		22 (31)
80–89		18 (26)
90–99		1 (1)
*Stage*		
TNM	AJCC *	
T1, T2-N0-M0	I	7 (10)
T3-N0-M0/T4-N0-M0	II	29 (41)
T1, T2-N1-M0/T3, T4-N1-M0	III	15 (21)
Any T-N2-M0/Any T-Any N-M1	IV	14 (20)
Not specified	Not specified	5 (7)

* AJCC: American Joint Committee on Cancer.

## Supplementary Materials

Supplementary materials can be found at https://www.mdpi.com/1422-0067/20/23/6082/s1. Figure S1. Expression patterns of Tpm3. Figure S2. Schematic image of the defined locations for tissue sample collection. Table S1. Antibodies used in this study. Table S2. List of identified polypeptides.
