# Supplementary material for: Gel-Based Proteomics of Clinical Samples Identifies Potential Serological Biomarkers for Early Detection of Colorectal Cancer
Source: Int J Mol Sci. 2019 Dec 2;20(23):6082. doi: 10.3390/ijms20236082 (PMC6929140; doi:10.3390/ijms20236082)
Supplement: Supplementary file 1 [file ijms-20-06082-s001.zip › supplementary material.docx]

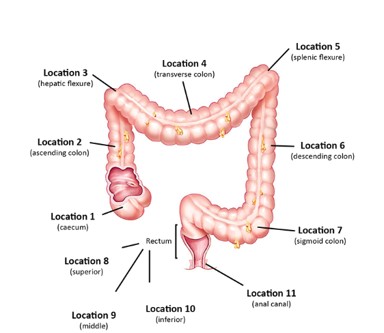


**Figure S1.** Schematic image of the defined locations for tissue sample collection.


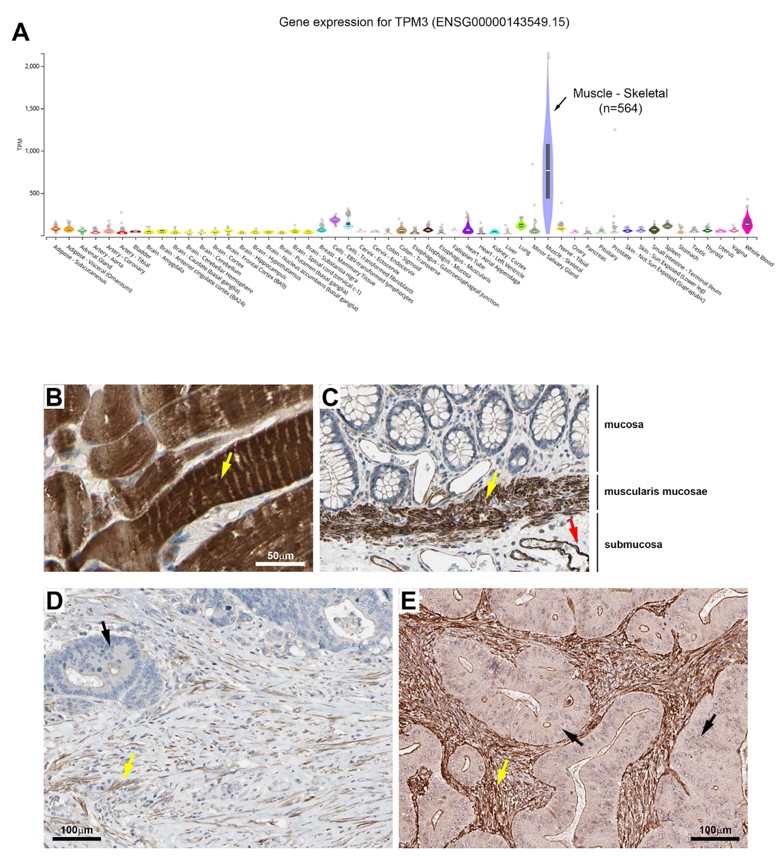


**Figure S2.** Expression patterns of Tpm3. (**A**) Expression of *TPM3* in various normal tissues. Expression values are shown in TPM (transcripts per million) calculated from a model with isoforms collapsed to a single gene [GTEx Analysis Release V7 (dbGaP Accession phs000424.v7.p2)]. IHC staining of Tpm3 protein in (**B**) skeletal muscle (yellow arrow; skeletal muscle), (**C**) normal colon tissue (yellow arrow, muscle layer; red arrow, blood vessel), and (**D** and **E**) different colorectal carcinoma samples (black arrow, tumor cells; yellow arrow, stromal cells). ([www.proteinatlas.org/pathology](http://www.proteinatlas.org/pathology); last accessed 05.03.2019). Magnification is provided in each case.

**Table S1. Antibodies used in this study.**

| **Protein Name** | **Short** | **Antibody Supplier** | **Catalog ID#1** | **UniProtKB** |
| --- | --- | --- | --- | --- |
| 78 kDa glucose-regulated protein^a,b^ | GRP78/HSPA5/BiP | RnD Systems | AF4846 | P11021 |
| α-enolase^a^ | Eno1 | Proteintech | 11204-1-AP | P06733 |
| Catalase^a,b^ | Cat (early 96-plex) | RnD systems | AF3398 | P04040 |
| Cyclophilin B^a,b^ | Scylp/PPIase B (early 96-plex) | RnD systems | AF5410 | P23284 |
| Eukaryotic translation elongation factor 2^a^ | eEF-2/EF-2 | RnD systems | AF3806 | P13639 |
| Galectin-3^a,b^ | Gal-3 | RnD systems | AF1154 | P17931 |
| Peroxiredoxin-1^a,b^ | Prdx1/Nkef-A (early 96-plex) | RnD systems | AF3488 | Q06830 |
| Profilin 1^a^ | Pfn1 | Proteintech | 11680-1-AP | P07737 |
| Receptor for activated C kinase 1^a,b^ | Rack1 (early96-plex) | RnD systems | AF3434 | P38011 |
| Tropomyosin alpha-3 chain^a,b^ | Tpm3/tm3 | SDIX | 2272.00.02 | P06753 |
